# Reducing the Frequency of Surveillance Blood Work in Patients Treated With Maintenance Hemodialysis: A Local Quality Improvement Initiative

**DOI:** 10.1177/20543581241255784

**Published:** 2024-05-28

**Authors:** Epsita Shome-Vasanthan, Sophia Chou, Juliya Hemmett, Jennifer MacRae, David Ward, Nathen Gallagher, Huda Al-Wahsh, Elena Qirjazi

**Affiliations:** 1 Faculty of Medicine & Dentistry, University of Alberta, Edmonton, Canada; 2 Cumming School of Medicine, University of Calgary, AB, Canada; 3 Alberta Kidney Care, Alberta Health Services, Calgary, Canada

**Keywords:** blood work, hemodialysis, anemia, mineral bone disease, quality improvement

## Abstract

**Introduction:**

There is little evidence on the ideal frequency of routine blood work in maintenance dialysis patients to manage complications, including anemia, mineral bone disease (MBD), and hyperkalemia. Recent quality improvement studies from Ontario showed no negative impacts when decreasing the frequency from monthly to every 6 weeks in conventional in-center hemodialysis (ICHD) patients. In December 2020, Alberta Kidney Care–South (AKC-S) reduced the frequency of routine blood work from every 6 weeks to every 8 weeks for ICHD patients.

**Objective:**

We aimed to assess the impact of reducing blood work frequency on patient outcomes.

**Methods:**

We compared prevalent AKC-S ICHD patients in 2 cohorts: (1) retrospective control (October 31, 2019-October 31, 2020) and (2) prospective intervention (December 1, 2020-December 1, 2021). Primary outcomes were true frequency of routine blood work, odds of patients being within target for anemia and MBD, and proportion of lab values of hyperkalemia. Furthermore, we compared hospitalizations and mortality.

**Results:**

A total of 972 patients in Calgary’s ICHD program were included, 787 in each period (with 602 patients overlapping both cohorts). The frequency of routine blood work decreased from every 39.5 days in the control period to every 54.2 days in the intervention period (*P* < .01). There was a reduction in the odds of phosphate values in targets (*P* = .02), and an increase in the odds of labs with hyperkalemia (>6.0 mmol/L) during the intervention period (*P* = .01). There was no significant change in the odds of being within the accepted targets during the intervention period compared with the control period for hemoglobin, Tsat, calcium, or parathyroid hormone (PTH). Fewer patients were hospitalized during the intervention period and the risk of death decreased as well, although additional factors such as the COVID-19 pandemic may have affected this. A cost-savings of $32 962 occurred from the reduced anemia and MBD blood work during the intervention period.

**Conclusions:**

When ICHD units in Calgary reduced routine blood work frequency from every 6 weeks to 8 weeks, there were no negative impacts on hospitalizations or deaths. A slightly lower proportion of phosphate values were within target, and a 0.7% increase in potassium values greater than 6 mmol/L was demonstrated. Our study suggests that blood work frequency in ICHD dialysis patients may be further reduced to every 8 weeks safely. Ultimately, additional pragmatic trials are needed to identify the optimal frequency of routine blood work.

## Abrégé

**Introduction:**

Il existe peu de données quant à la fréquence idéale des analyses sanguines chez les patients sous dialyse d’entretien pour gérer les complications comme l’anémie, les troubles liés à la densité osseuse (TDO) et l’hyperkaliémie. De récentes études d’amélioration de la qualité menées en Ontario n’ont montré aucune conséquence négative lorsque la fréquence des analyses des patients traités de façon traditionnelle par hémodialyse en centre (HDC) est passée d’une fois par mois à une fois toutes les 6 semaines. L’Alberta Kidney Care-South (AKC-S) a réduit la fréquence des analyses sanguines de routine; depuis décembre 2020, les analyses des patients traités par HDC sont effectuées toutes les 8 semaines plutôt qu’aux 6 semaines.

**Objectif:**

Évaluer les effets d’une réduction de la fréquence des analyses sanguines sur les résultats des patients.

**Méthodologie:**

Nous avons comparé des patients de l’AKC-S prévalents pour l’HDC dans deux cohortes: a) période témoin — rétrospective (31 octobre 2019 au 31 octobre 2020); b) période d’intervention — prospective (1^er^ décembre 2020 au 1^er^ décembre 2021). Les principaux critères d’évaluation étaient la fréquence réelle des analyses sanguines de routine, la probabilité que les patients soient dans la cible pour l’anémie et les TDO, et dans la fourchette cible des valeurs de laboratoire pour l’hyperkaliémie. Les hospitalisations et la mortalité ont également été comparées entre les deux cohortes.

**Résultats:**

Au total, 972 patients du programme d’HDC de Calgary ont été inclus, soit deux cohortes de 787 patients (602 patients ont chevauché les deux cohortes). Les analyses sanguines étaient effectuées tous les 39,5 jours pendant la période témoin; cette fréquence est passée aux 54,2 jours pendant la période d’intervention (p<0,01). On a observé une réduction des probabilités que le taux de phosphate soit dans les cibles (p=0,02) et une augmentation des valeurs de laboratoire montrant une hyperkaliémie (>6,0 mmol/L) pendant la période d’intervention (p=0,01). Aucun changement significatif n’a été observé entre les deux périodes en ce qui concerne la probabilité que les valeurs de laboratoire soient dans les cibles acceptées pour l’hémoglobine, le TSAT, le calcium ou la PTH. Pendant la période d’intervention, moins de patients ont été hospitalisés et le risque de décès a diminué, mais d’autres facteurs, notamment la pandémie de COVID-19, ont pu influer sur ce résultat. Enfin, le fait de réduire la fréquence des analyses de suivi pour l’anémie et les TDO a entraîné des économies de 32 962 $.

**Conclusion:**

Aucune conséquence négative n’a été observée lorsque la fréquence des analyses sanguines des patients des unités d’hémodialyse en centre de Calgary est passée de 6 à 8 semaines. Une proportion légèrement plus faible des valeurs de phosphate se situait dans la fourchette cible et une augmentation de 0,7 % des valeurs de potassium supérieures à 6 mmol/L a été constatée. Notre étude suggère qu’il est sécuritaire d’effectuer les analyses sanguines des patients hémodialysés en centre toutes les 8 semaines. Des essais pragmatiques sont nécessaires pour déterminer la fréquence optimale de ces analyses.

## What was known before

Optimal routine blood work frequency in patients treated with maintenance dialysis is not known, with conventional blood work frequency generally being once a month. Recent studies showed that reducing blood work frequency from every 4 weeks to every 6 weeks was safe and achieved similar outcomes. There are pros and cons to frequent surveillance blood work for complications of chronic kidney disease, such as anemia, mineral bone disease, or hyperkalemia.

## What this adds

When in-center hemodialysis units in Calgary reduced routine blood work frequency from every 6 weeks to 8 weeks, there were no negative impacts on hospitalizations or deaths. A slightly lower proportion of phosphate values were within target, and a 0.7% increase in potassium values greater than 6 mmol/L was demonstrated. The long-term clinical impacts and relevance of these findings are unclear, and ultimately, additional pragmatic trials are needed to compare optimal frequency of testing.

## Introduction

Patients with chronic kidney disease (CKD) are at risk of many complications, including anemia and mineral bone disease (MBD). These complications are monitored with laboratory tests (hemoglobin [Hgb] and iron saturation [Tsat] for anemia; parathyroid hormone [PTH], calcium, and phosphate for MBD).^1,2^ The optimal frequency of this surveillance blood work in dialysis patients (CKD5D) is unknown. Society guidelines from Kidney Disease Improving Global Outcomes (KDIGO) and the Canadian Society of Nephrology (CSN) have ungraded recommendations from monthly to every 3 months surveillance blood work for CKD5D.^3,4^

Frequent laboratory testing provides more opportunities to intervene and potentially achieve recommended targets. However, when patients are already at target, the utility of more frequent blood work is limited, particularly for MBD.^5^ Furthermore, there are consequences to frequent blood work, including significant expenses to the health care system and potential negative impacts on patients, such as worsening anemia, false positives, anxiety, and time allotment.^6^

Two prior quality improvement studies done by Thomas et al^7^ and Silver et al^8^ based in Ontario showed that after decreasing the frequency of routine blood work from every 28 to 31 days to every 52 days (6 weeks) in patients on conventional in-center hemodialysis (ICHD), there were no negative impacts on achieving anemia and MBD targets, or episodes of hyperkalemia, nor a difference in hospitalizations or cardiovascular death. Furthermore, Silver et al^8^ calculated cost-savings of $85 CAD per patient year, and estimated yearly savings of $35 000 CAD for a 400-patient hemodialysis center.

In addition, due to the COVID-19 pandemic, laboratory services (including Alberta Laboratory Services, Alberta Precision Labs [APL]), have been overwhelmed due to implemented measures of social distancing and increased COVID-19 testing. The APL asked for more judicious use of blood tests by physicians. In this setting, Alberta Kidney Care–South (AKC-S) implemented a quality improvement initiative with the objective of reducing routine blood work frequency in maintenance ICHD patients in Calgary, Alberta, from every 6 to every 8 weeks over 1 year. The effect of this change on patient outcomes was evaluated by assessing hospitalizations and mortality, as well as biochemical outcomes (achieving anemia and MBD targets, and hyperkalemia). We hypothesized that this change would be safe for patients and would not result in significant differences in clinical and biochemical results.

## Methods

### Study Design

This was a prospective cohort quality improvement study aimed to assess the impact of reduced routine laboratory testing in patients treated with maintenance ICHD. In the AKC-S program in Calgary (7 hemodialysis units), routine blood work frequency was reduced from every 6 weeks to every 8 weeks as of December 1, 2020.^9^ The intervention period was defined from December 1, 2020, to December 1, 2021 (every 8-week blood work), with a control period for comparison from October 31, 2019, to October 31, 2020 (every 6-week blood work). Throughout both time periods, physicians were able to order any additional blood work as clinically indicated at their discretion.

### Routine

Blood work days were counted when patients had all of the following labs drawn: complete blood count (CBC), electrolytes, creatinine, urea, calcium, albumin, and phosphate. Otherwise, *total* lab days were counted when any (but not all) of the lab values of interest (Hgb, electrolytes, Tsat, Calcium, Phosphate, etc) were drawn.

### Ethics

This study was submitted to the Project Ethics Community Consensus Initiative (ARECCI) screening tool, attaining a score of 1—low risk for participants.^10^ The study proposal was subsequently submitted to the University of Calgary, Alberta, Health Research Ethics Board and, given its low risk and quality improvement methodology, it was exempted from formal research ethics board review on November 25, 2020.

### Patient Population

All adult (18 years or older) patients in AKC-S Calgary, who had been on maintenance ICHD for at least 3 months during the two 12-month periods, were included.

### Data Collection

The AKC-S Patient-based Renal Information System (PARIS) is a database for all renal patients in the program. This database contains demographic data, hemodialysis prescriptions, blood work results (including timing), and data around hospitalizations, death, and modality changes. The PARIS was used to extract patient baseline characteristics—including age, sex, and presence of diabetes mellitus and cardiovascular disease (a composite of coronary artery disease, heart failure, and/or peripheral vascular disease)—these comorbidities are documented and inputted by AKC-S staff. Furthermore, the database was also used to extract hospitalizations, mortality, blood work frequency, and results that are directly acquired from other databases (such as APL and Clinibase).

### Outcomes

We assessed the uptake of this intervention by measuring the frequency of routine blood work and total lab days for patients. Outcome measures included the odds of patients’ lab values being within target for anemia (Hgb and Tsat), and MBD (calcium, phosphate, and PTH) (Table 2), as well as proportion of hyperkalemia (defined as potassium greater than 6.0 mmol/L). Balancing measures assessing the clinical safety in patients included hospitalizations, patient mortality, rates of renal transplant, and transfers to home dialysis. Lastly, cost-savings were calculated based on APL costs of individual tests for anemia and MBD (Table 2).

**Table 1.** Definition of Routine and Total Blood Work Days.

	Definition	Methodology
Routine blood work day	A day when **all of**: CBC, electrolytes, creatinine, urea, calcium, albumin, and phosphate were drawn	Changed from q6weeks to q8weeks
Total blood work day	**Any** day a single or multiple lab tests (but not all) were drawn including: CBC, electrolytes, creatinine, urea, calcium, albumin, and/or phosphate	No changes imposed between control and intervention periods

**Table 2.** Local APL Costs and AKC-S Targets (Based on Clinical Guidelines and Local Practices) for Laboratory Tests Studied for Anemia and MBD Targets.

Lab test	Cost ($CAD)	AKC-S target
Hemoglobin	7	95-110 g/L
Iron saturation	5	0.2-0.4
Calcium	5	2.1-2.6 mmol/L
Phosphate	5	1.1-1.8 mmol/L
Parathyroid hormone	9	100-400 ng/L

**Table 3.** Cohort Baseline Characteristics.

Characteristic	
Count of patients during the two 12-month periods	972
Count of patients during the control period	787
Count of patients during the intervention period	787
Age (years, mean [SD])	63.6 (15.0)
Sex	
Female %	367 (37.8)
Male %	605 (62.2)
*Comorbidities*	
Cardiovascular disease	
With cardiovascular disease (%)	446 (45.9)
Without cardiovascular disease (%)	526 (54.1)
Diabetes (%)	
With diabetes (%)	572 (58.8)
Without diabetes (%)	396 (40.7)
Missing (%)	4 (0.4)

### Statistical Analysis

All analysis was conducted using R software, version 4.1.2. Data were analyzed using descriptive statistics (counts and percentages for categorical variables, means and standard deviations or medians and interquartile ranges for continuous ones). Mixed regression models were used to account for the correlation between observations from the same patient. Logistic regression mixed models, including random intercept for patients, were used to assess the effect of reducing routine blood work frequency from every 6 weeks to every 8 weeks on the odds of achieving anemia and MBD targets, with a separate model fitted for each marker. Similarly, a logistic regression mixed model was used to assess the odds of hyperkalemia events.

Zero-inflated mixed negative binomial model, including random intercept for patients, was used to estimate the effect on the number of hospitalization days of the patient per year. Zero-inflated negative binomial model was used to manage overdispersion and excess zeros in the number of hospitalization days. Logistic regression mixed model compared the proportion of patients without hospitalization days (zero hospitalization days) in the intervention period to the proportion of patients without hospitalization days in the control period.

Extended Cox regression model using time-dependent variables was used to assess the effect on the risk of death, and to account for the correlation between observations from the same patient. All the models were adjusted for age, sex, routine lab days, total lab days, diabetes, and cardiovascular disease. Two-sided *P* value <.05 was considered statistically significant.

## Results

### Patient Demographics

A total of 972 patients in Calgary’s ICHD program were included, with 787 patients in each of the control and intervention periods, and 602 (61.9%) included in both cohorts. This significant overlap of patients makes statistical comparisons of baseline characteristics futile. In the overall cohort, the mean age was 63.6 (SD = 15.0) years; 367 (37.8%) were women; 572 (58.8%) had diabetes mellitus, and 446 (45.9%) had cardiovascular disease (coronary artery disease, congestive heart failure, or peripheral vascular disease).

### Frequency of Blood Work

The frequency of routine blood work decreased from every 40 days during the control period to every 51 days during the intervention period, reflecting adequate uptake of the protocol within the hemodialysis units (*P* < .001). Despite this true reduction, the frequency of total lab days remained similar during the 2 time periods, every 12 days in the control period compared with every 13 days in the intervention period (*P* = .16).

### Blood Work Targets

**Figure 1.**
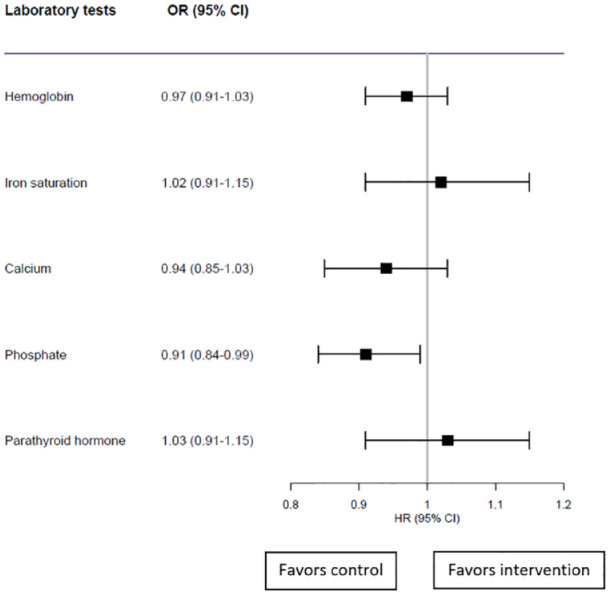
Odds ratios of being within targets during the intervention period compared with the control.

There was no significant change in patients’ odds of being within the accepted targets during the intervention period compared with the control period for hemoglobin, Tsat, calcium, or PTH (Figure 1). The only exception was for phosphate, where the odds of being within target during the intervention decreased by 9% (95% confidence interval [CI] = 0.84-0.99) (*P* = .02).

### Hyperkalemia

In the control period, 6.8% of 14 617 potassium tests were greater than 6 mmol/L, compared with 7.5% of 12 568 tests in the intervention. The absolute increase in percentage of tests with hyperkalemia was therefore 0.7%. Adjusted logistic mixed regression model showed that the odds of hyperkalemia (defined as potassium >6 mmol/L) during the intervention period was significantly increased by 18% (95% CI = 1.04-1.33) (*P* = .01). Further analysis showed that in the control period, 0.98% of potassium tests were greater than 7 mmol/L (critical hyperkalemia), compared with 1.26% in the intervention period. The absolute increase in percentage of tests with critical hyperkalemia was therefore 0.28%.

### Hospitalizations and Exit From Cohort

There was no significant difference in the number of hospitalization days per patient in the intervention period compared with the control period (95% CI = 0.70-1.05) (*P* = .13). Fewer patients were hospitalized during the intervention period and the odds of *not* being hospitalized during the intervention period were 2.3 times higher (95% CI = 1.81-2.81) (*P* < .01).

Table 4 shows exits from the cohort in the form of mortality, rates of transplantation, and transfers to home dialysis modalities in the 2 study periods. Mortality significantly decreased during the intervention period with a hazard ratio of 0.28 (95% CI = 0.16-0.50) (*P* < .01).

**Table 4.** Patients Who Exited the Cohort Early, Categorized Based on Reasons: Death, Kidney Transplantation, or Transferring to Home Dialysis.

Exit from cohort	Control (%)	Intervention (%)
Death	85 (10.8)	82 (10.4)
Kidney transplantation	45 (5.7)	32 (4.1)
Home dialysis	25 (3.2)	23 (3.0)

### Costs

Based on current APL costs for anemia and MBD labs, the total cost of all labs done during the control and intervention period was determined. A total cost-savings of $32 962 CAD was calculated during the intervention period from reduced anemia and MBD blood work frequency.

## Discussion

After reducing blood work frequency in patients treated with maintenance ICHD from every 6 weeks to every 8 weeks, we did not see worse clinical outcomes such as hospitalizations or mortality. In terms of biochemical outcomes, there was a reduction in the odds of patients’ phosphate values being within target, but not the other anemia and MBD markers. There was also a statistically significant increase in hyperkalemia tests, although the absolute difference was less than 1% and the clinical significance is unclear. Our results differ from the previous quality improvement studies from Ontario. Our study pushed the interval between routine blood work even further (8 weeks instead of 6 weeks). To our knowledge, there are no studies assessing the impact of reducing routine blood work frequency from 4 weeks to 8 weeks. The increased interval may explain the differences in phosphate and potassium values within target, which were not ascertained previously.

Our finding of decreased odds of phosphate values within target when blood work frequency is reduced is difficult to interpret. Abnormal phosphate has been shown to be an independent marker of mortality in patients treated with maintenance dialysis.^5,11^ A systematic review showed that the risk of death increased by 18% for every mg/dL increase in phosphate level, and a cohort study in Australia and New Zealand observed a U-shaped association between phosphate and all-cause mortality in these patients.^12^ Even so, there are no studies showing improved mortality with treating abnormal phosphate and frequent monitoring may not result in normalized phosphate. Yokoyama et al^13^ showed that weekly measurements were not associated with achieving phosphate targets in ICHD patients. Therefore, the clinical significance of our phosphate findings is unclear, particularly as no increase in mortality was seen in our study. In addition, this finding could have been a consequence of confounding factors, such as dietary changes and the COVID-19 pandemic.

Hyperkalemia was increased by 0.7% and 0.28% for potassium values greater than 6 and 7 mmol/L respectively—a concern given the risks of arrhythmia and cardiac arrest.^14–19^ Patients with predialysis hyperkalemia have a higher incidence of all-cause and cardiovascular mortality; predialysis potassium values between 4.6 and 5.3 were associated with the greatest survival in patients on maintenance hemodialysis.^14,19^ Even so, given we found no increase in mortality, clinical significance is unclear. Furthermore, while the frequency of routine blood work did decrease, the number of blood work days (which often consisted of individual tests such as predialysis potassium when patients feel unwell) stayed the same in our study periods. As such, it is unclear whether increased *routine* blood work would result in improved potassium levels.

Our clinical outcomes of interest, hospitalizations and mortality, did not increase during the intervention period. However, this study took place during the COVID-19 pandemic, with multiple potential confounding factors. Pandemic fluctuations may have affected the rates of AKC-S patients accessing or avoiding hospital-care. Data from the Canadian Institute for Health Information found that between March 2020 and June 2021, there were fewer emergency department visits compared to prepandemic.^20^ Ultimately, the lack of rise in mortality and hospitalizations when blood work frequency was reduced is a reassuring signal for the safety of this intervention, although long-term follow-up and additional studies would be required to confirm this finding.

Strengths of our study include its prospective design, and very few exclusion criteria (only incident dialysis patients who had been on dialysis less than 3 months were excluded to allow for stability). Furthermore, there was significant overlap in the patient population between the control and intervention period, as 62% of patients continued maintenance dialysis therapy through the 2 years allowing for continuity in assessing any impacts from reducing blood work frequency. We had good uptake with regard to reducing routine blood work frequency, making our results more accurate. Last, by focusing on ICHD patients, we also captured a patient population that is the most comorbid and at highest risk of adverse outcomes such as hospitalizations and mortality.

Nevertheless, our study has several limitations. First, data extraction was limited to PARIS. Lab values for all anemia and MBD targets were categorized as either being within the AKC-S target or outside of it. As such, even naturally occurring high hemoglobin (without use of erythropoietin-stimulating agents) were classified as outside our targets. While this may have underestimated the patients achieving anemia targets, we have no reason to suspect any systematic difference between the 2 study periods. Furthermore, data available for causes of death and hospitalization were not reliable. In addition, 1 year may be too short of a follow-up time to detect significant clinical changes. Last, given the observational design, confounding factors could not be eliminated. Specifically, the control period started prior to the COVID-19 pandemic while the intervention period was completely during the pandemic. There were many possible factors that changed throughout these time periods, including health care avoidance patterns and vaccination availability.

In conclusion, our prospective study reduced routine blood work frequency within Calgary’s ICHD units and resulted in significant cost-savings. We found no negative impacts on patient outcomes such as hospitalizations or mortality. Overall, these findings add to the growing data that standard every 4 to 6 weeks routine blood work in patients treated with maintenance hemodialysis may be unnecessary, particularly given the lack of evidence supporting optimal blood work frequency.

Future directions include confirming safety of reducing routine blood work frequency, ideally through pragmatic randomized controlled trials across many geographic locations, or at minimum additional observational studies as suggested by Lacson and Meyer.^21^ Furthermore, studying patient and staff perceptions is key for the uptake and sustainability of integrating any further changes in policy around routine blood work frequency.
